# Supplementary material for: The interactome of intact mitochondria by cross-linking mass spectrometry provides evidence for coexisting respiratory supercomplexes
Source: Mol Cell Proteomics. 2017 Dec 8;17(2):216–32. doi: 10.1074/mcp.RA117.000470 (PMC5795388; doi:10.1074/mcp.RA117.000470)
Supplement: Supplemental Data [file supp_RA117.000470_133922_0_supp_23978_fzffwf.zip › spectra_annotation/mito_DR_spectra_annotation/150-1-6-1-8-1.pdf]

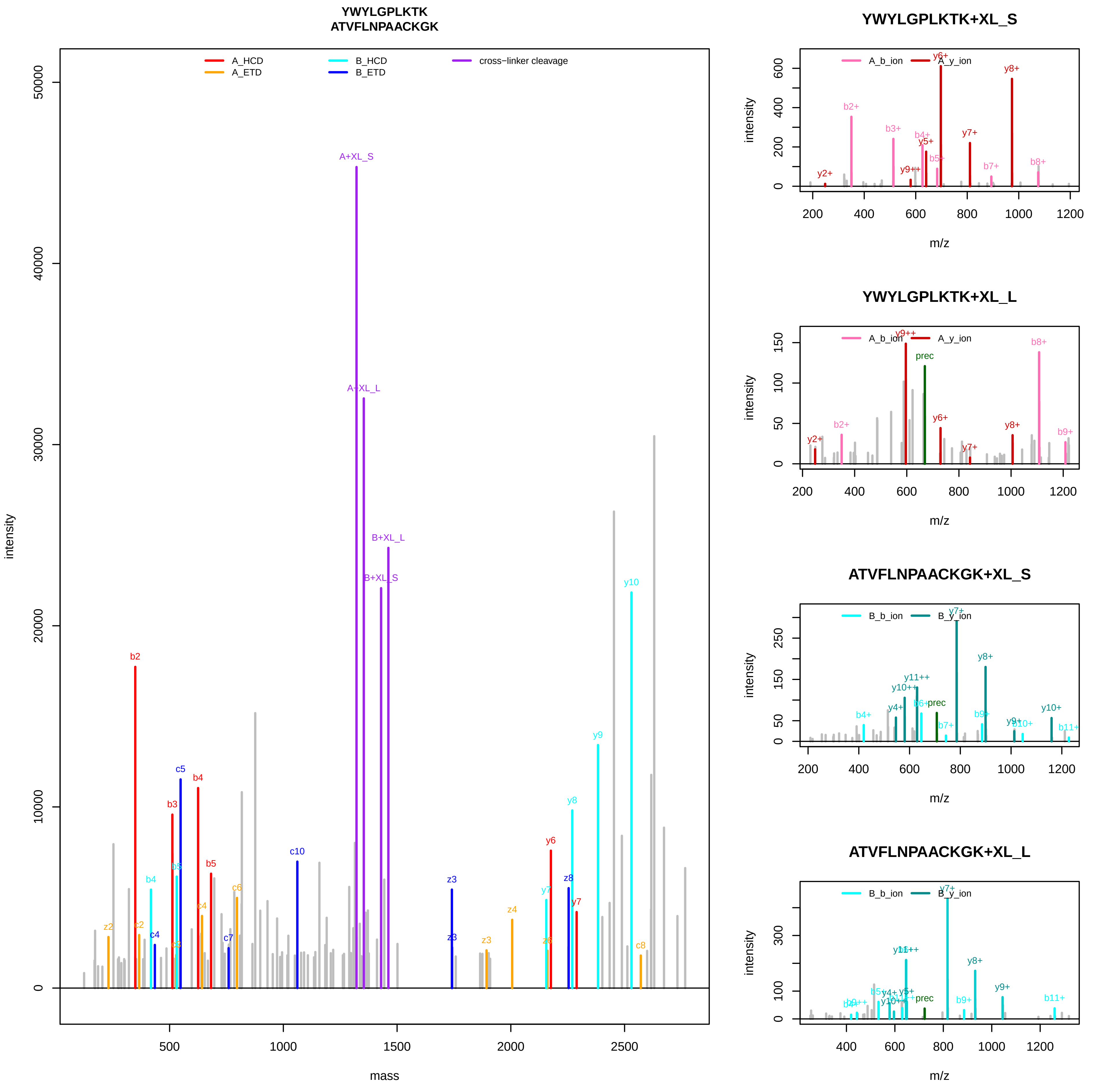
YWYLGPLKTK
ATVFLNPAACKGK
A_HCD
A_ETD
B_HCD
B_ETD
cross−linker cleavage
intensity
mass
0
10000
20000
30000
40000
50000
500
1000
1500
2000
2500
A+XL_S
A+XL_L
B+XL_L
B+XL_S
b2
b3
b4
b5
b4
b5
c2
c3
c4
c5
c6
c7
c8
c10
z2
z3
z4
z6
z8
y6
y7
y8
y9
y10
YWYLGPLKTK+XL_S
A_b_ion
A_y_ion
b2+
b3+
b4+
b5+
b7+
b8+
y2+
y5+
y6+
y7+
y8+
y9++
YWYLGPLKTK+XL_L
b2+
b8+
b9+
y2+
y6+
y7+
y8+
y9++
prec
ATVFLNPAACKGK+XL_S
B_b_ion
B_y_ion
b4+
b6+
b7+
b9+
b10+
b11+
y4+
y7+
y8+
y9+
y10+
y10++
y11++
prec
ATVFLNPAACKGK+XL_L
b4+
b5+
b9+
b11+
y4+
y5+
y7+
y8+
y9+
y11++
prec
m/z
intensity
